# *Streptacidiphilus toruniensis* sp. nov., isolated from a pine forest soil

**DOI:** 10.1007/s10482-016-0759-5

**Published:** 2016-08-24

**Authors:** Patrycja Golinska, Hanna Dahm, Michael Goodfellow

**Affiliations:** 1 Department of Microbiology, Nicolaus Copernicus University, 87 100 Torun, Poland; 2 School of Biology, University of Newcastle, Newcastle upon Tyne, NE1 7RU UK

**Keywords:** Actinobacteria, Pine forest soil, Polyphasic taxonomy, *Streptacidiphilus toruniensis*

## Abstract

Two acidophilic actinobacteria, isolates NA14 and NF37^T^, were the subject of a polyphasic taxonomic study. Chemotaxonomic and morphological properties of the isolates were characteristic of the genus *Streptacidiphilus*. The isolates were shown to have identical 16S rRNA gene sequences and to be closely related to *Streptacidiphilus neutrinimicus* DSM 41755^T^ (>99.9 %). However, DNA:DNA relatedness between isolate NF37^T^ and the type strain of *S. neutrinimicus* was found to be low at 11.1 (±3.5) %. A broad range of phenotypic features were shown to distinguish the isolates from their close phylogenetic neighbours. These data shown that the isolates form a novel species of *Streptacidiphilus* for which the name *Streptacidiphilus toruniensis* sp. nov. is proposed. The type strain is NF37^T^ (= DSM 102291^T^ = NCIMB 15025^T^).

**Electronic supplementary material:**

The online version of this article (doi:10.1007/s10482-016-0759-5) contains supplementary material, which is available to authorized users.

## Introduction

The genus *Streptacidiphilus* was proposed by Kim et al. (2003) who classified it in the family *Streptomycetaceae* along with the genera *Kitasatospora* and *Streptomyces*. The taxonomic status of the genera *Kitasatospora* and *Streptacidiphilus* have been questioned (Kämpfer 2012a; Labeda et al. 2012); though extensive molecular systematic data support the continued recognition of the former as a separate genus (Ishikawa et al. 2010; Girard et al. 2014), corresponding studies on the genus *Streptacidiphilus* are awaited.

Streptacidiphili are currently assigned to ten species with validly published names most of which have been circumscribed in extensive polyphasic studies (Golinska et al. 2013a, b). *Streptacidiphilus* strains grow between pH 3.5 and 6.0, optimally around pH 5.0, form extenstively branched substrate hyphae which differentiate into flexuous to straight chains of smooth-surfaced spores, contain major proportioins of LL-diaminopimelic acid in whole-organism hydrolysates, saturated, iso- and anteiso fatty acids, hexa- and octahydrogenated menaquinones with nine isoprene units as predominant isoprenologues, and complex polar lipid patterns that include diphosphatidylglycerol, phosphatidylethanolamine, phosphatidylinositol and phosphatidylinositol mannosides (Kämpfer 2012a, b). Streptacidiphili are common and widely distributed in acidic habitats, notably in coniferous soils (Williams et al. 1971; Golinska et al. 2013a, b), produce chitinases and diastases with low pH optima (Williams and Flowers 1978), are a source of antifungal compounds (Williams and Khan 1974) and are implicated in the turnover of organic matter at low pH values (Goodfellow and Williams 1983; Williams et al. 1984).

In a continuation of our investigations on the diversity of acidophilic sporoactinomycetes in acid forest soils, several strains were isolated that had colonial properties typical of streptacidiphili. Two of these strains, isolates NA14 and NF37^T^, were included in a polyphasic taxonomic study which showed that they represent a novel *Streptacidophilus* species for which we propose the name *Streptacidiphilus toruniensis* sp. nov.

## Materials and methods

### Sampling site

Soil samples were collected from a pine forest on the northern slope of an inland sand dune in the Torun Basin, Poland (52^o^55′ 37″N, 18^o^42′11″E) in October 2013. The trees were planted in 1875. The pine needles formed a well defined horizon which was divided into three layers; the L layer which consisted of about 3 cm of intact needles, a 3 cm deep F layer of partially decomposed but recognisable needles and a 2.5 cm deep H layer of a black-brown amorphous mass of decomposed needles, humic material and other organic matter. The underlying A1 horizon was 8 cm deep. The mean pH of the three litter layers and the mineral horizon were 4.4, 4.0, 3.7 and 3.7, respectively.

### Organisms, maintenance and biomass preparation

Acidophilic filamentous sporoactinobacteria were sought from the L, F, H and A1 horizons of the pine forest soil by spreading aliquots (100 µl) of serial dilutions over the surfaces of oven dried plates of starch – casein agar (Kűster and Williams 1964) supplemented with cycloheximide and nystatin (each at 50 µg ml^−1^) and adjusted to pH 4.5 with 1 N HCl. The inoculated plates were incubated at 28 °C for 4 weeks, after which *Streptacidiphilus*-like colonies were detected from suspensions prepared from each of the environmental samples. Nine of the isolates were found to grow on starch-casein agar at pH 4.5 but not at pH 7.5.

The isolates were maintained on acidified starch-casein agar slopes at room temperature and as suspensions of mycelial fragments and spores in glycerol (20 %, v/v) at −80 °C. Strains NF37^T^ and NA14 which were isolated from the F and A1 horizons, respectively, were chosen for detailed taxonomic analyses together with the type strains of *Streptacidiphilus durhamensis*, *Streptacidiphilus hamsterleyensis* and *Streptacidiphilus neutrinimicus*.

Biomass for the molecular systematic and most of the chemotaxonomic studies was prepared by cultivating the two isolates in shake flasks of acidified yeast extract-malt extract (ISP2; International *Streptomyces* Project medium 2, Shirling and Gottlieb 1966) broth (pH5.5), at 150 revolutions per minute at 28 °C for 2 weeks. Cells were harvested by centrifugation and washed twice in distilled water; biomass for the chemotaxonomic analyses was freeze dried and that for the molecular systematic work stored at −20 °C. Biomass (~40 mg) for the fatty acid analysis carried out on isolate NF37^T^ was prepared by scraping growth from plates of ISP2 agar, adjusted to pH 5.5, following incubation at 28 °C for 7 days.

### Phylogenetic analyses

Extraction of genomic DNA, PCR-mediated amplification of 16S rRNA genes of the two isolates and direct sequencing of the purified PCR products were carried out as described by Golinska et al. (2013c). The closest phylogenetic neighbours based on 16S rRNA gene sequence similarities were found using the EzTaxon server (http://eztaxon-e.ezbiocloud.net/, Kim et al. 2012). The resultant 16S rRNA gene sequences were aligned with those of the type strains of *Streptacidiphilus* species using Clustal W. Phylogenetic analyses were carried out using MEGA 6 (Tamura et al. 2013) and PHYML (Guindon and Gascuel 2003) software packages following multiple alignment using Clustal W. Evolutionary distances were calculated and clustering determined using the maximum-likelihood, maximum-parsimony and neighbour-joining methods and the resultant tree topologies evaluated by a bootstrap analysis (Felsenstein 1985) of the neighbour-joining method based on 1000 resamplings using the MEGA 6 software. The trees were rooted using the 16S rRNA gene sequence of *Streptomyces albus* subsp. *albus* DSM 40313^T^ (GenBank accession number AJ621602).

### DNA:DNA relatedness

DNA:DNA relatedness values (∆Tm) between isolate NF37^T^ and *S. neutrinimicus* DSM 41755^T^ were determined in duplicate at the DSMZ (Braunschweig, Germany). Cells were disrupted by using a Constant System TS 0.75 KW machine (IUL Instruments, Germany). DNA was isolated using a French pressure cell (Thermo Spectronic) and purified using a hydroxyapatite column, as described by Cashion et al. (1977). DNA–DNA hybridizations were carried out as described by De Ley et al. (1970), with the modifications described by Huss et al. (1983), using a model Cary 100 Bio UV/VIS spectrophotometer equipped with a Peltier-thermostatted 6 × 6 multicell changer and a temperature controller with an in situ temperature probe (Varian) at 71 °C.

### Chemotaxonomy

Isolates NF37^T^ and NA14 were examined for chemotaxonomic markers considered to be of value in the systematics of genera classified in the family *Streptomycetaceae* (Kämpfer 2012a). To this end, standard chromatographic methods were used to determine the isomers of diaminopimelic acid (Staneck and Roberts 1974), isoprenoid quinones (Collins 1985), polar lipids (Minnikin et al. 1984) and whole-organism sugars (Hasegawa et al. 1983), using appropriate controls. Cellular fatty acids extracted from isolate NF37^T^ were methylated following the procedure described by Miller (1982) with minor modifications from Kuykendall et al. (1988). The fatty acid methyl esters were separated by gas chromatography (Hewlett Packard instrument 6890 N) and the resultant peaks automatically integrated, fatty acid names and percentages were determined using the standard Microbial Identification MIDI System; version 5 (Sasser 1990). The TSBA40 version of the MIDI database was used to identify the fatty acids. The G + C mol % of the DNA of the isolate was determined by HPLC following the procedure described by Tamaoka and Komagata (1984). Purified DNA prepared as described earlier was hydrolysed with P1 nuclease, the nucleotides dephosphorylated with bovine alkaline phosphatase (Mesbah et al. 1989) then analysed by HPLC (Shimadzu Corp., Japan). Lambda-DNA and three DNAs with published genome sequences representing a G + C range of 43–72 mol % were used as standards. G + C values were calculated from the ratio of deoxyguanosine and deoxythymidine, after Mesbah et al. (1989).

### Cultural and morphological properties

The isolates were examined for cultural and morphological features following growth on acidified (pH 5.5) ISP media 1–7 (Shirling and Gottlieb 1966), as described previously (Golinska et al. 2013a). Hyphal and spore chain configuration were detected on acidified oatmeal agar (ISP medium 3; Shirling and Gottlieb 1966) after 14 days at 28 °C, using the coverslip technique of Kawato and Shinobu (1959). Spore arrangement and spore surface ornamentation of isolate NF37^T^ were established by examining a gold-coated dehydrated preparation from the acidified oatmeal agar plate with a scanning electron microscope (Model 1430 VP, LEO Electron Microscopy Ltd, Cambridge, England) using the procedure described by O’Donnell et al. (1993).

### Phenotypic tests

An extensive range of phenotypic tests were carried out on the isolates using media and methods described by Williams et al. (1983), albeit with acidified media. The ability of the isolates to grow at various temperatures (4, 10, 15, 20, 25, 30, 35 and 40 °C), pH values 4.0–7.5 at 0.5 pH intervals and NaCl concentrations (1, 3, 5, 7 and 10 %, w/v) were determined using acidified ISP2 agar (Shirling and Gottlieb 1966), apart from the temperature tests all of the media were incubated at 28 °C for 3 weeks. The results of these tests were compared with corresponding data for the type strains of *S. durhamensis*, *S. hamsterleyensis* and *S. neutiminicus* which had been acquired using the same media and methods. The enzymatic profiles of the isolates and the type strains of their nearest neighbours were acquired using API ZYM kits (Bio Mérieux) according to the manufacturer’s instructions.

## Results

### 16S rRNA gene sequencing and DNA:DNA relatedness studies

**Fig. 1:**
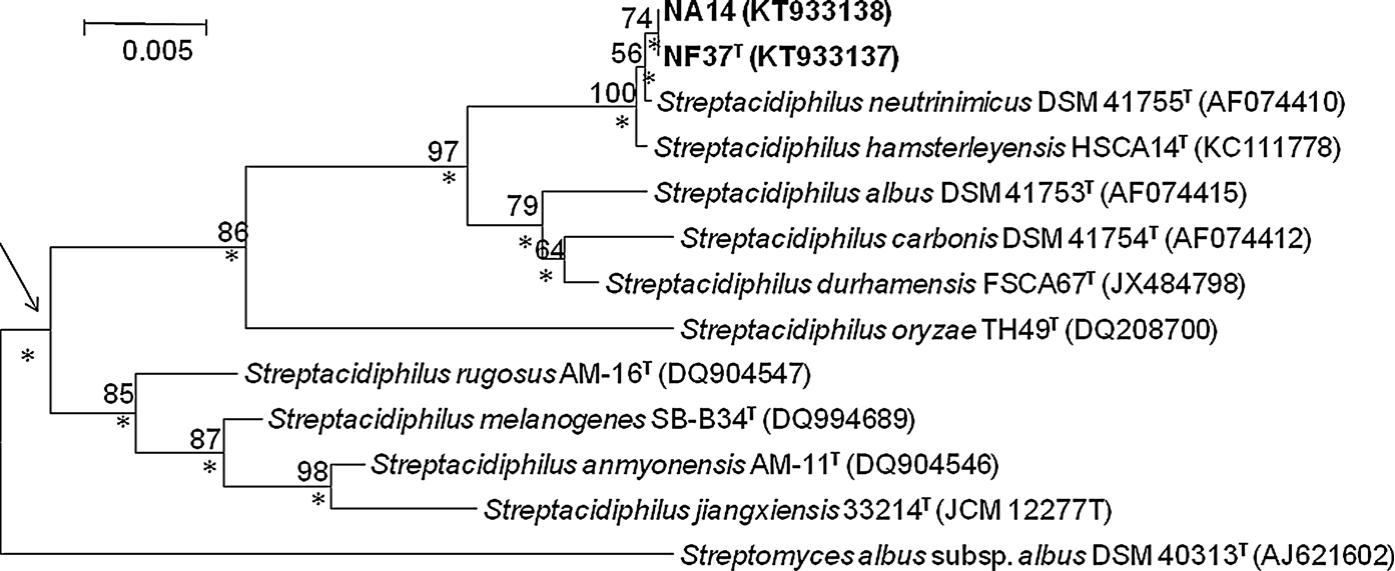
Neighbour-joining tree based on nearly complete 16S rRNA gene sequences (1376–1511 nucleotides) showing relationships between the isolates and between them and the type strains of *Streptacidiphilus* species. *Asterisks* indicate branches of the tree that were also found using the maximum-likelihood and maximum-parsimony tree-making algorithms. Numbers at the nodes are percentage bootstrap values based on 1000 re-sampled datasets, only values above 50 % are given. T, type strain. *Bar* 0.005 substitutions per nucleotide position. The root position of the tree was determined using *Streptomyces albus* subsp. *albus* DSM 40313^T^ as the outgroup

Nearly complete 16S rRNA gene sequences of the isolates (1382–1393 nucleotides [nt]) NF37^T^ and NA14 were determined. The strains were found to have identical sequences (Genbank accession numbers: KT933137 and KT933138, respectively) and to form a branch in the *Streptacidiphilus* 16S rRNA gene tree together with the type strains of *Streptacidiphilus albus*, *Streptacidiphilus carbonis*, S. *durhamensis*, *S. hamsterleyensis* and *S. neutrinimicus* (Fig. 1); this cluster was shown to be supported by a 97 % bootstrap value and by all of the tree-making algorithms. Isolates NA14 and NF37^T^ were shown to be closely related to *S. neutrinimicus* DSM 41755^T^ sharing a 16S rRNA gene sequence similarity with the latter of 99.9 %, a value found to correspond to 1 nt difference. The corresponding 16S rRNA gene sequence similarities between isolates NF37^T^ and NA14 with the type strains of *S. hamsterleyensis*, *S. durhamensis*, *S. albus* and *S. carbonis* were shown to be 99.9 and 99.9 %, 98.7 and 98.7 %, 98.6 and 98.6 % and 98.3 and 98.3 %, respectively. The 16S rRNA gene sequence similarities with the type strains of the remaining *Streptacidiphilus* species fell within the range 95.8–96.8 %.

The DNA:DNA similarity value between isolate NF37^T^ and *S. neutrinimicus* DSM 41755^T^ was shown to be 11.1 (±3.53)  %, a result well below the 70 % cut-off point recommended for the assignment of closely related strains to the same genomic species (Wayne et al. 1987).

### Chemotaxonomy

Isolates NA14 and NF37^T^ were shown to have whole-organism hydrolysates rich in LL-diaminopimelic acid; galactose and rhamnose, contained major proportions of tetra-, hexa- and octahydrogenated menaquinones with nine isoprene units and in ratios of 1.0:2.5:2.4 and 1.0:6.1:11.5, respectively, and diphosphatidylglycerol, phosphatidylethanolamine (diagnostic marker), phosphatidylglycerol, phosphatidylinositol and phosphatidylinositol mannosides, as predominant polar lipids (see online Fig. S1). The cellular fatty acid profile of isolate NF37^T^ was shown to consist of major proportions (>12 %) of *anteiso*-C_15:0_ (19.4 %), *iso*-C_16:0_ (15.4 %), C_16:0_ (16.4 %), *anteiso*-C_17:0_ (12.6 %), low proportions (>1.3 %) of *iso*-C_14:0_ (2.0 %), *iso*-C15:0 (6.9 %), *iso*-C16:1 H (1.4 %), *iso*-C_17:1_ ω9c (1.5 %), *anteiso*-C_17:1_ ω9c (2.1 %), *iso*-C_17:0_ (2.0 %), C_17:0_ cyclo (4.6 %) plus trace amounts of other components (<1 %). The genomic G + C content of isolate NF37^T^ was 72.3 mol %.

### Phenotypic properties

The isolates were found to have many phenotypic features in common, some of which distinguished them from their close phylogenetic neighbours (Table 1). The isolates, unlike the type strain of *S. neutrinimicus*, their nearest phylogenetic neighbour, produced a white aerial spore mass and moderate/strong yellow substrate mycelia on ISP7 medium and grew at 30 °C. In contrast, the *S. neutrinimicus* strain, unlike the isolates, was found to grow at pH 3.5, used L-arabitol and D-xylitol as sole carbon sources and displayed chymotrypsin activity. In addition, the isolates were found to have a greater capacity to grow on sole carbon and sole nitrogen sources than the type strains of *S. durhamensis* and *S. hamsterleyensis*. Additional phenotypic features of the isolates are cited in the species description.

**Table 1:** Phenotypic properties that distinguish the isolates from the type strains of their nearest taxonomic neighbours

Characteristics	Isolates NA14 and NF37^T^	*Streptacidiphilus durhamensis* DSM 45796^T^	*Streptacidiphilus hamsterleyensis* DSM 45900^T^	*Streptacidiphilus neutrinimicus* DSM 41755^T^
Growth on tyrosine agar (ISP 7)				
Aerial spore mass	White	Light gray	Light gray	Light gray
Substrate mycelium	Moderate/strong yellow	Moderate yellowish brown	Light yellowish brown	Strong yellowish brown
Hydrolysis of: aesculin	+	+	–	+
Arbutin	+	+	–	+
Reduction of nitrate	–	–	+	–
Degradation of starch	+	+	–	+
Growth on sole carbon sources at 1 %, w/v				
L-arabitol	–	–	–	+
Dextran	+	–	–	+
*Meso*-erythritol	+	–	–	+
*Meso*-inositol	+	–	–	+
D-salicin	+	+	–	+
Xylitol	–	–	–	+
at 0.1 %, w/v				
Sodium citrate	+	–	–	+
Sodium fumarate	+	–	–	+
Sodium hippurate	+	–	–	+
Sodium oxalate	+	–	–	+
Sodium succinate	+	–	–	+
Growth on sole nitrogen sources (0.1 %, w/v)				
l-aspartic acid	+	–	–	+
l-cysteine	+	–	–	+
l-*iso*leucine	+	+	–	+
l-phenylalanine	+	+	–	+
l-valine	+	+	–	+
API-ZYM tests				
α-chymotrypsin	–	–	–	+
α-glucosidase	+	+	–	+
*N*-acetyl-β-glucosaminidase	+	+	–	+
Valine arylamidase	+	–	–	+
pH range of growth	4.0–6.5	4.5–6.0	4.5–6.0	3.5–5.5
Temperature range growth (°C)	10–30	10–30	10–30	10–25
G + C (mol%)	72.3	71.0	71.0	71.0

+ positive; − negative

Isolates NA14 and NF37^T^ shared many properties in common but were found to produce distinctive aerial spore mass and substrate mycelial colours on some of the ISP media (Table 2), whilst only the former grew on adonitol, d-fructose and D-trehalose as sole carbon sources and on l-arginine and l-asparagine as sole nitrogen sources. In turn, isolate NF37^T^ produced β-glucosidase and α-mannosidase (API ZYM tests).

**Table 2:** Growth and cultural characteristics of the isolates and their closest phylogenetic neighbours on ISP media after incubation for 3 weeks at 28 °C

ISP medium	NF37^T^			NA14			*Streptacidiphilus durhamensis* DSM 45796^T^			*Streptacidiphilus hamsterleyensis* DSM 45900^T^			*Streptacidiphilus neutrinimicus* DSM 45755^T^		
	Growth	Aerial spore mass colour when formed	Colour of substrate mycelium	Growth	Colour of aerial mass	Colour of substrate mycelium	Growth	Aerial spore mass colour when formed	Colour of substrate mycelium	Growth	Aerial spore mass colour when formed	Colour of substrate mycelium	Growth	Aerial spore mass colour when formed	Colour of substrate mycelium
1	+	–	Beige	+	–	Pale greenish yellow	+	White	Light yellow	+	Light gray	Grayish yellow	+	None	Beige
2	+++	None	Light yellow	+++	Light greenish gray	Dark yellowish brown	++	Light gray	Yellowish brown	+++	Medium gray	Yellowish brown	**++**	White	Moderate yellow
3	++	Light Gray	Pale greenish yellow	++	Yellowish grey	Deep yellowish brown	+++	Medium gray	Light grayish yellowish brown	++	White to gray	Dark orange yellow	++	White	Pale yellow
5	+++	White scant	Light greenish yellow	+++	None	Light yellow	++	Light gray	Moderate yellowish brown	+++	Light gray	Light yellowish brown	++	White	Moderate yellow
6	+	None	Beige	+	None	Pale yellow	–	None	None	–	None	None	+	None	Pale yellow
7	+++	White	Moderate yellow	+++	White	Strong yellow	+	Light gray	Moderate yellowish brown	++	Light gray	Light yellowish brown	+++	White	Strong yellowish brown

None of the strains formed diffusible pigment on any of the ISP media, except strain NA14 which produced a deep yellow pigment on ISP medium 2

Only isolate NA14 grew on ISP 4 producing a scant greenish yellow substrate mycelium

+++ abundant growth, ++ moderate growth, + poor growth,− no growth

## Discussion

Filamentous sporactinomycetes which grow between pH 3.5 and 6.0 are a neglected group even though they are common in acidic habitats (Williams et al. 1971; Khan and Williams 1975; Goodfellow and Dawson 1978; Golinska et al. 2013a, b, 2015). The present study provides further evidence that the genus *Streptacidiphilus* is underspeciated (Lonsdale 1985; Seong et al. 1993, 1995) as isolates NA14 and NF37^T^ were found to belong to a new *Streptacidiphilus* species for which the name *Streptacidiphilus toruniensis* sp. nov. is proposed. Comparative taxonomic surveys of streptacidiphili isolated from neglected and unexplored acidic habitats can be expected to throw additional light on the extent of the taxonomic diversity encompassed by the genus *Streptacidiphilus*.

### Description of *Streptacidiphilus toruniensis* sp. nov

*Streptacidiphilus toruniensis*. N.L. masc. adj. *toruniensis*, belonging to the Polish city of Torun, the source of the isolates.

**Fig. 2:**
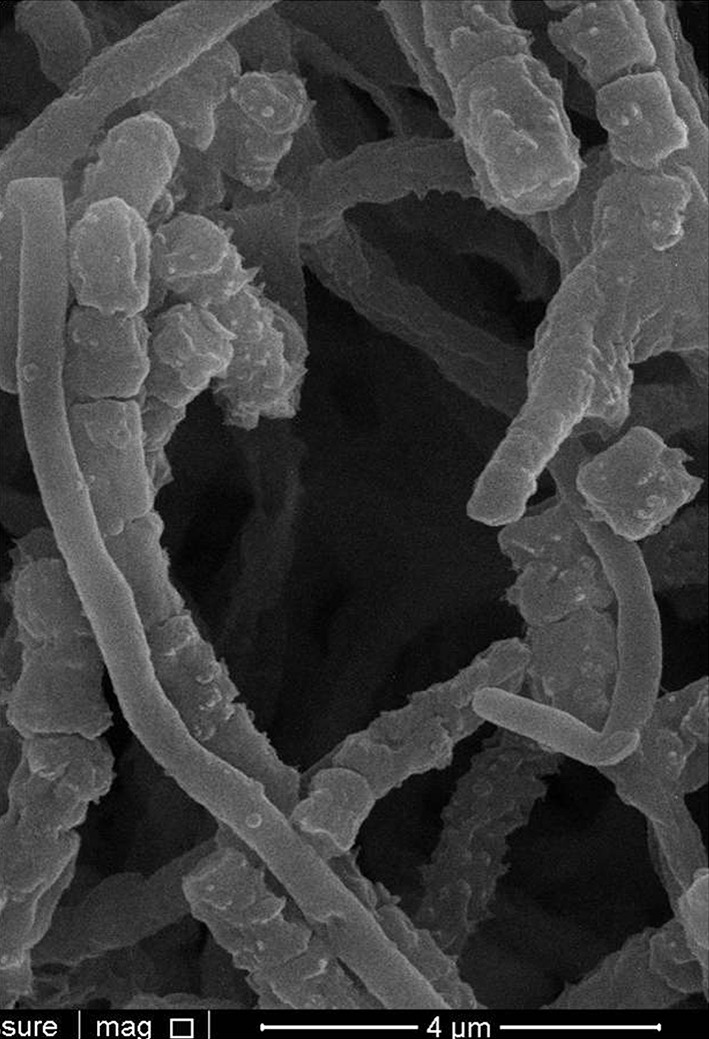
Scanning electron micrograph of isolate NF37^T^ showing straight chains of smooth-surfaced, cylindrical spores on oatmeal agar after growth for 3 weeks at 28 °C. Bar, 4 µm

Aerobic, Gram-positive, non-acid alcohol fast, acidophilic actinobacteria which form an extensively branched substrate mycelium that carries aerial hyphae which differentiate into long straight to flexuous chains of smooth, cylindrical spores (Fig. 2) (0.6 × 0.9 µm). Grows from 10 to 30 °C, optimally ∽27 °C, from pH 4.5 to 6.5, optimally ∽pH 5.5, and in the presence of 1 %, but not at 3 % NaCl (w/v). Degrades Tweens 40 and 60, but not adenine, casein, chitin, elastin, gelatin, guanine, hypoxanthine or l-tyrosine. l-arabinose, d-cellobiose, d-galactose, d-glucosamine, d-glucose, glycogen, d-lactose, d-maltose, d-melibiose, β-methyl-D-glucoside, d-raffinose, d-rhamnose, d-salicin and d-xylose are used as sole carbon sources for energy and growth, but not d-glucuronic acid (all at 1 %, w/v). Neither acetate, adipate, benzoate, butyrate and propionate (sodium salts) nor *para*-hydroxybenzoic acid are used as sole carbon sources (all at 0.1 %, w/v). l-alanine, l-hydroxyproline, l-serine, and l-threonine are used as sole nitrogen sources, but not l-methionine (all at 0.1 %, w/v). Additional phenotypic properties are given in Tables 1 and 2. The major fatty acids of the type strain are *anteiso*-C_15:0_, *iso*-C_16:0_, C_16:0_, *anteiso*-C_17:0_ The remaining chemotaxonomic markers are typical of the genus *Streptacidiphilus*. The G + C content of the DNA of the type strain is 72.3 mol %.

The type strain is NF37^T^ (= DSM 102291^T^ = NCIMB 15025^T^) and strain NA14 is a second strain. These strains were isolated from the F and A1 horizons of a *Pinus sylvestris* L. forest in the Torun Basin, Poland.

## Electronic supplementary material

Below is the link to the electronic supplementary material.

Supplementary material 1 (TIFF 543 kb)
