# Supplementary figures and images for: Streptacidiphilus toruniensis sp. nov., isolated from a pine forest soil
Source: Antonie Van Leeuwenhoek. 2016 Aug 24;109(12):1583–91. doi: 10.1007/s10482-016-0759-5 (PMC5104812; doi:10.1007/s10482-016-0759-5)

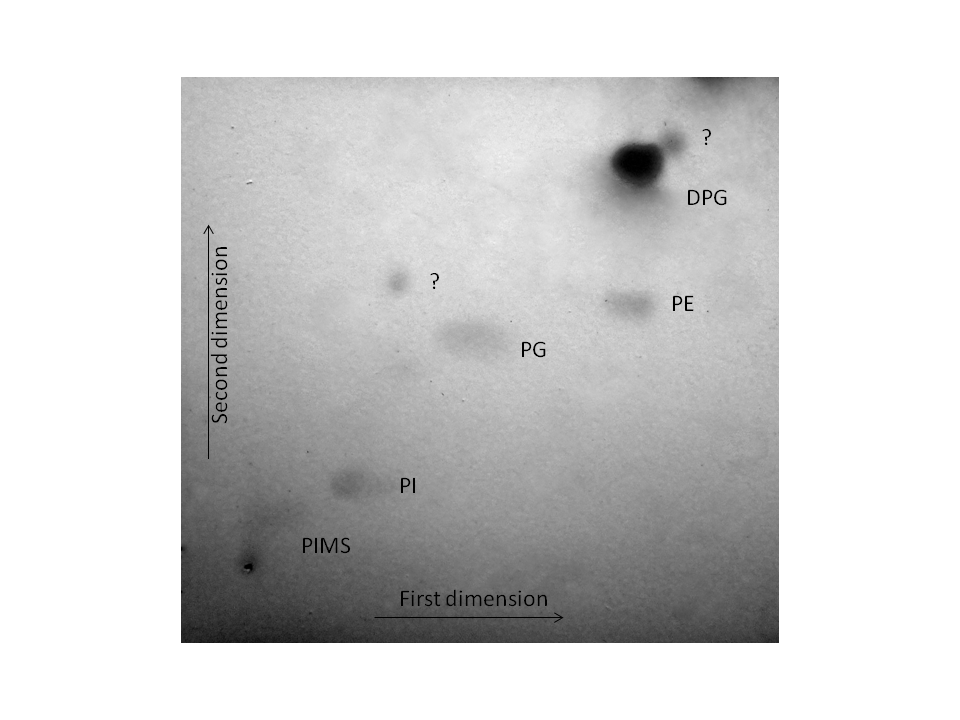

Supplement: Supplementary file 1 — Supplementary material 1 (TIFF 543 kb) [file 10482_2016_759_MOESM1_ESM.tif]
